# The Duty of Young Men to the Profession

**Published:** 1901-03

**Authors:** Guy R. Danforth


### THE DUTY OF YOUNG MEN TO THE PROFESSION.[1]

BY GUY R. DANFORTH, D.D.S.

THE subject I have chosen for a brief summary this afternoon may appear to some of you as being familiar. I will admit that the subject, as well as some parts of the subject-matter, was taken from a well-known work,—viz., "The Practice Builder," by Dr. C. R. Hambly.

The late Collis P. Huntingdon said, in his advice to young men, "No one can follow in the footsteps of another. He must work out his own destiny." This, I think, holds good in the dental profession as well as in any other calling in life. A young man beginning the practice of dentistry has, or, if not, he should have, an ideal, a worthy example of success, some man who by a noble character and by conscientious and devoted attention to his profession

---

[1] Read at the sixth annual meeting of the Jefferson County Dental Society, Watertown, N. Y.

has attained a high standard of proficiency. He should consider at the start that "anything worth doing is worth doing well."

A young man may work his way through a dental college, pass the scheduled examinations of the course, and manage to squeeze through the final tests. He goes into active practice, either for himself or with some older practitioner, without even daring to try the examination required by the State in which he is practising. Does this man do his duty? Is he honest in the first steps of his professional career? A young man's duty to self is an all-important factor in reckoning his future success. He should, to the best of his ability, become familiar with theoretical dentistry before leaving college, and then, by close application, study, and careful practice of theory, become a thorough master of practical dentistry.

After the performance of duty to one's self in the line of professional education and qualifications follows a very important duty,—that of a young practitioner to his patient. An operation about the mouth, no matter how trivial it may seem, must be performed with extreme care and under the most strict aseptic conditions. It does not take years of experience to find that the least neglect or carelessness on your part in the treatment or filling of a tooth may, and almost invariably does, result in disaster. The best is none too good, and in some cases an operation performed with the greatest thoroughness will result in failure. Thus we see that under no circumstances whatever must a young practitioner neglect, to even the slightest degree, his part in the preservation of a tooth to which his professional attention has been called.

Every one is liable to mistake, but when one's services are needed it is his duty to do the very best in his judgment in the particular case brought to his attention. The greatest success is achieved by the man who strives to do the very best work that can be done, no matter how long it takes him or how little his recompense may be.

A young dentist is apt to have a large variety of patients. The "shoppers" always give him a call. The "dissatisfied" call to have him examine the plate they are wearing or some filling they have had done,—"to get your opinion," they say. The best reply to such might be, "that you are not in the habit of passing criticism on other people's work." There are also charity patients, which if worthy should receive attention. But the most desirable and certainly the best patients are his friends who are anxious for his success. The others should be treated with due respect, but not catered to.

The main object of a young man in beginning the practice of his profession is to please. There are many ways in which to do this; in fact, as many ways as there are classes of people. But I presume to say that the quickest and surest way to please the largest number of people would be to put the price of gold crowns down to three dollars, plates to five dollars, and other work accordingly. I would feel better satisfied, I believe, to please a small number of people for a long period of time than to please a large number of people a short time.

It is the natural tendency of people to go where they can get the most for the least money. This is especially true in dentistry. But the question is, Do these people profit in the end?

Every one knows—if they do not, they should know—that the organs of mastication are as valuable to them as any part of their anatomy, and that the cost of their preservation should not be considered. It pays in the end to use the very best material that can be obtained, and take the greatest possible pains in every case, even if the recompense at that time is not as great as you would desire. The price should be such that you can afford to use the best material and spend any amount of time necessary for the completion of the work. "Economy is a good quality, but one may use too much of it and lose money." "That economy which has for its purpose the very best materials that money can buy, without variation, irrespective of maker or seller, is the only economy that can be practised in dentistry. If the economy that is based upon liberal conceptions of your obligation to your patients is practised, it cannot but result to your profit. A young man who desires that his patrons should have confidence in him must have confidence in himself. He may be troubled with a lack of confidence or over-confidence, both of which are fatal. It is better to know that you do not know than to believe you know when you do not know." (Hambly.)

Honesty is one of the first stepping-stones towards the attainment of success. It is claimed by many that "Dishonesty is the best business policy." It cannot be denied but that some succeed in this way, but a solid and more permanent foundation can be built by strict honesty than by any amount of misrepresentation.

An all-important factor in the conduct of a young man is his respect for his brother dentist. It should be courteous, dignified, and gentlemanly. You are our fathers or our brothers. The very least that you can expect of us who are younger in years and experience is that we shall prove to every one of you that we are gentlemen. No dentist knows what moment he may have need for the friendship of another. At the very moment when he least expects it he may need evidence in a law-suit as to his ability.

"A young man should scrupulously and strenuously avoid the *rôle* of a cynic."

A cynic, as you all know, is one who never sees a good quality in a brother of the same profession. "He is a human owl, vigilant in darkness, but blind to the light. He goes mousing for vermin and never seeks noble game. His criticisms fall indiscriminately upon the old and young like frost upon flowers. If a man is said to be noble and good, he will answer, 'Yes, in the daytime.' If his neighbor is having a good practice, he says, 'Yes, people want their pay.' If he joins the lodge or the church, he says, 'Yes, to get practice.' Such a man is generous, 'Yes, with other people's money.' This man is very obliging, 'Yes to lull suspicion and cheat you.' Thus his eye strains out every good quality and takes in only the bad." As I think of my fellow-man and my brother in the profession, I like to think that something has survived the fall, something of good lives in him. Man has been called "A desolate city, and his passions are like the wild beasts of the wilderness howling in kings' palaces." Yet, he is God's workmanship, and a thousand touches of exquisite beauty yet remain. "A man will be what his most cherished feelings are."

If he will encourage a noble generosity, a spirit of manliness and honor and true fraternity towards the profession, every feeling of his own soul will be enriched; but if he shall nurse bitter and envenomed thoughts, his own spirit will absorb the poison; he then will be the loser and he will crawl among men as a burnished adder whose life is mischief and whose errand is death.

"He who hunts for flowers will find flowers, and he who loves weeds will find weeds." Honesty, manliness, temperance, integrity, industry, and economy are words every young man should seek to be familiar with, for in these words are whole volumes from which a young man may learn his duty to the profession.

